# Premature and accelerated aging: HIV or HAART?

**DOI:** 10.3389/fgene.2012.00328

**Published:** 2013-01-28

**Authors:** Reuben L. Smith, Richard de Boer, Stanley Brul, Yelena Budovskaya, Hans van Spek

**Affiliations:** Swammerdam Institute for Life Sciences, University of AmsterdamAmsterdam, Netherlands

**Keywords:** mitochondria, HIV, HAART, antiretroviral, *C. elegans*, immunosenescence, premature and accelerated aging, NRTI

## Abstract

Highly active antiretroviral therapy (HAART) has significantly increased life expectancy of the human immunodeficiency virus (HIV)-positive population. Nevertheless, the average lifespan of HIV-patients remains shorter compared to uninfected individuals. Immunosenescence, a current explanation for this difference invokes heavily on viral stimulus despite HAART efficiency in viral suppression. We propose here that the premature and accelerated aging of HIV-patients can also be caused by adverse effects of antiretroviral drugs, specifically those that affect the mitochondria. The nucleoside reverse transcriptase inhibitor (NRTI) antiretroviral drug class for instance, is known to cause depletion of mitochondrial DNA via inhibition of the mitochondrial specific DNA polymerase-γ. Besides NRTIs, other antiretroviral drug classes such as protease inhibitors also cause severe mitochondrial damage by increasing oxidative stress and diminishing mitochondrial function. We also discuss important areas for future research and argue in favor of the use of *Caenorhabditis elegans* as a novel model system for studying these effects.

## HIV-INFECTION

**FIGURE 1:**
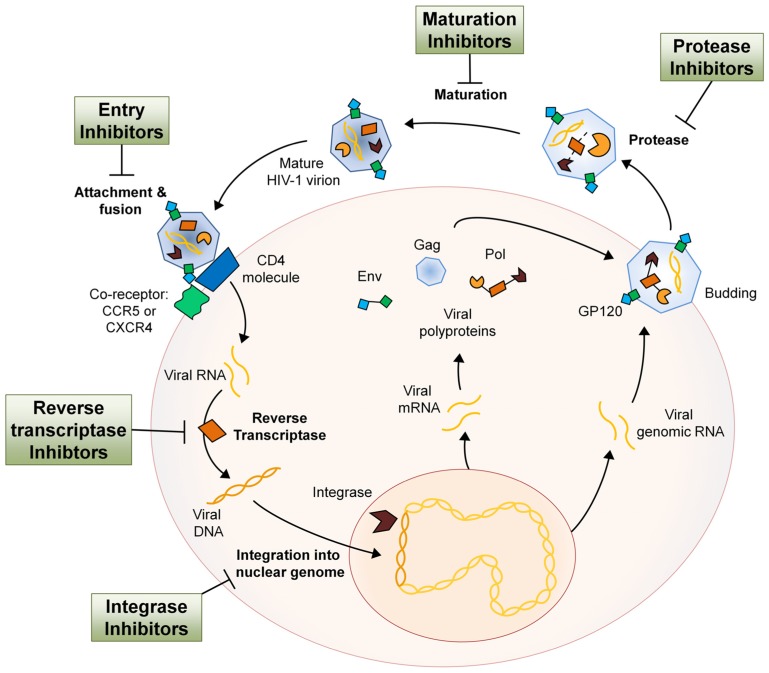
**The HIV-1 life cycle and the antiretroviral drug class intervention points**. Entry inhibitors interfere with viral entry into the host cell and are comprised of a complex group of drugs with multiple mechanisms of action. By inhibiting several key proteins that mediate the process of virion attachment, co-receptor binding and fusion, virus spreading can be mitigated (Tilton and Doms, 2010). NRTIs imitate endogenous deoxyribonucleotides and have a high affinity for the viral reverse transcriptase, thus facilitating incorporation into the viral DNA strand during synthesis. NRTI incorporation results in transcription termination as they all lack the 3′-OH group necessary for phosphodiester bond formation in DNA strand elongation (Cihlar and Ray, 2010). NNRTIs are compounds that fit into the allosteric “pocket” site of the HIV-1 reverse transcriptase and disrupt its enzymatic activity, selectively blocking HIV-1 transcription (De Clercq, 2004). Integrase inhibitors bind cofactors of the viral integrase that are essential in host DNA interaction and therefore block insertion of proviral DNA into the host genome (Schafer and Squires, 2010). Protease inhibitors bind the viral protease active site with high affinity and therefore inhibit cleavage of viral polypeptides and subsequent maturation of the virion after budding from the host cell (Adamson, 2012). HIV-1 maturation inhibitors act much like protease inhibitors in that they inhibit the processing of the HIV-1 polypeptides. However, maturation inhibitors do not bind the protease but rather the polypeptide itself, rendering it uncleavable (Richards and McCallister, 2008). The relative size of different components has been altered for pictorial clarity.

The human immunodeficiency virus (HIV-1) is a *Retrovirus* of the *Lentivirus* genus that primarily infects cells of the host immune system. Once an individual is infected, HIV-1 replication takes place in several steps. In the first step, the virion attaches itself to the host cell with the help of co-receptors, whereupon it fuses with the host cell membrane and the two single-stranded RNA molecules and three different viral enzymes are released into the host cell cytoplasm. The viral reverse transcriptase transcribes the viral RNA into DNA, at which point the viral DNA is transported into the nucleus. With the aid of the viral integrase the viral DNA is processed and incorporated into the host genome. The integrated viral DNA, now known as a provirus, is transcribed and translated by the host machinery to synthesize viral proteins and single-stranded RNA for new virions. After assembly of these components at the plasma membrane, the new virions bud off and mature using the viral protease, completing the HIV-1 life cycle (**Figure 1;**Teixeira et al., 2011). HIV infection of host immune cells causes them to die and thus drastically deplete in number. As immune cell counts decline, the host gradually becomes immune-incompetent and more susceptible to opportunistic infections. If untreated, this leads to acquired immune deficiency syndrome (AIDS) and eventually death.

## ANTIRETROVIRAL THERAPY

For the treatment of HIV-1 infection there are currently six different classes of anti-HIV drugs. Each class of drug acts on a particular aspect of the viral life cycle (**Figure 1**), and are used in unison to increase therapy efficacy, overcome problems of tolerance, and decrease emergence of viral resistance. The major classes include the entry inhibitors (EIs), the nucleoside reverse transcriptase inhibitors (NRTIs), the non-nucleoside reverse transcriptase inhibitors (NNRTIs), and the protease inhibitors (PIs). The additional two anti-HIV drug classes are the maturation inhibitors (MIs) and integrase inhibitors (IIs), of which most compounds are still in clinical development.

Since 1996 the combination of at least three antiviral drugs, preferably from at least two different classes, has become standard practice and is known as highly active antiretroviral therapy (HAART). Due to the large variety in drug combinations, standard HAART has been defined as one or more NRTIs combined with a PI (**Table 1**) and often supplemented with one drug from another class (Dybul et al., 2002). Due to the replicative speed of HIV-1 and the inability of antiretroviral drugs to eradicate infection, patients need to medicate daily for the rest of their lives. Nonetheless, the therapeutic use of a combination of drugs was a major advance in HIV therapy and has significantly improved the quality and length of patient lives.

**Table 1:** Antiretroviral drugs discussed in this review.

Antiretroviral drug class	Drug name	Other names/abbreviations
Nucleoside reverse transcriptase inhibitor (NRTI)	Alovudine	FLT (3′-deoxy-3′-fluorothymidine)
	Didanosine	ddI (2′,3′-dideoxyinosine)
	Stavudine	D4T (2′,3′-didehydro-2′,3′-deoxythymidine)
	Zalcitabine	ddC (2′,3′-dideoxycytidine)
	Zidovudine	AZT (3′-azido-3′-deoxythymidine)
Protease inhibitor (PI)	Indinavir	IDV
	Lopinavir	LPV
	Nelfinavir	NFV
	Ritonavir	RTV
	Saquinavir	SQV

## HAART TREATED HIV-PATIENTS AGE PREMATURELY

Without antiretroviral therapy HIV-infected patients usually die within years because of immune system failure. Due to HAART however, early death is prevented, allowing HIV-patients to live decades as long medication is continued (May and Ingle, 2011). It was recently estimated that more than 50% of HIV-infected patients in the United States will be over the age of 50 in 2015 (Effros et al., 2011). Even though this gain in lifespan is celebrated as a success, data show that the life expectancy of treated patients remains shorter than that of the normal population (The Antiretroviral Therapy Cohort Collaboration, 2008). Life expectancy for treated HIV-patients is dependent on the age at which antiretroviral therapy is started and is estimated to be 10–30 years less than that of the uninfected (Lohse et al., 2007). Several studies have also observed that co- and multi-morbidities, like cardiovascular disease, diabetes, and osteoporosis, which are normally witnessed later on in life as a result of natural aging, were increasingly prominent among the HIV-infected population (Deeks and Phillips, 2009; Guaraldi et al., 2011). These observations led to the hypothesis that the HAART treated HIV-infected population is aging more rapidly, a phenomenon now known as premature and accelerated aging.

## THEORIES FOR PREMATURE AND ACCELERATED AGING IN HAART TREATED PATIENTS

There are several factors that influence lifespan of the HIV-infected, but have limited effects on progression of premature and accelerated aging phenotypes. These include lifestyle risk factors such as smoking, drinking, and illicit drug use, which are prevalent across the HIV-infected population (Shurtleff and Lawrence, 2012). Illicit drug use for example, is associated with poorer medication adherence and lesser immunological and virological control (Lucas et al., 2001). Additionally, co-infection, such as with viral hepatitis, is common among the HIV-infected population and is known to decrease life expectancy (Sulkowski, 2008). HIV-1 patients also run a greater risk for adverse drug interactions due to the increase in “pill-burden” to combat co-morbidities (Marzolini et al., 2011). Moreover, both natural aging or HIV-1 infection cause changes in gastrointestinal tract, liver, and kidney function that collectively affect the pharmacology of administered drugs (McLean and Le Couteur, 2004). None of these factors however can directly be related to causing the premature and accelerated aging phenotype witnessed in treated HIV-patients (Martin and Volberding, 2010).

Most research in this relatively new field focuses on how HIV-1 infection depletes CD4^+^ cell counts and exhausts the patient’s immune system (Appay and Sauce, 2008; Desai and Landay, 2010). In this way, HIV-infection itself if left untreated has been shown to convert the immune system of a young individual into one similar to someone 40 years older (Ferrando-Martínez et al., 2011). This theory of an accelerated aging process of the immune system is called immunosenescence and is characterized by continuous immune provocation and systemic low-grade inflammation, which predisposes patients to co-morbidities and natural aging symptoms more frequently seen in the elderly (Dock and Effros, 2011; Deeks et al., 2012).

The immunosenescence theory of aging has substance when considering untreated patients, as it principally focuses on viral effects. However, this theory is less plausible for treated patients as HAART has proven highly successful in swiftly replenishing CD4^+^ cell counts and reducing viral-load to barely detectable limits (Camacho and Teófilo, 2011). Additionally, various antivirals have been shown to induce inflammatory signals and it is therefore plausible that if an altered immune-organization is seen in HAART treated patients it is due to antiretroviral therapy (Mondal et al., 2004; Lagathu et al., 2007; Lefèvre et al., 2010). The influence HAART has warrants thorough investigation as HIV-patients take HAART daily and for the rest of their lives. Very few premature and accelerated aging studies in the HIV-infected population however, focus upon the influence that antiretroviral drugs have on aging and age-related co-morbidities. Accordingly, no consensus has arisen as to why the successfully treated HIV-infected population shows signs of premature and accelerated aging.

## IS HAART THE PREDOMINANT CAUSE OF PREMATURE AND ACCELERATED AGING?

Antiretroviral therapy as an explanation for premature and accelerated aging was first mentioned in studies wherein clinical symptoms of aging were shown to correlate with adverse side effects of antiretroviral therapy (Onen et al., 2010). For example, cardiovascular disease, diabetes, kidney and liver disease, metabolic disorders, osteoporosis, and lipodystrophy have all been associated with HAART (Effros et al., 2011; Klein, 2011). Accelerated Tau deposition, a marker for neurodegenerative diseases such as Alzheimer’s and Parkinson’s, has also been shown to be elevated in patients receiving HAART compared to HIV-infected non-treated patients (Anthony et al., 2006). These symptoms collectively seem to be related to tissues with high-energy demand and show a strong similarity to hereditary mitochondrial diseases (Schapira, 2012). Indeed, after introduction of HAART to treat HIV-1 infection, it quickly became apparent that mitochondrial toxicity is a major reason for antiretroviral-related adverse events (Brinkman et al., 1998). HAART-induced mitochondrial dysfunction therefore likely plays a role in most, if not all complications associated with premature and accelerated aging (White, 2001; Hulgan and Gerschenson, 2012). The specific influence of HAART upon mitochondria and aging however, is often not addressed.

## HAART-RELATED MITOCHONDRIAL TOXICITY IN AGING

Mitochondria are essential organelles in the life cycle and fitness of the cell. They are principal regulators of apoptosis and ATP production. Mitochondria are also involved in calcium and reactive oxygen species (ROS) homeostasis. Therefore, a perturbation of any of these functions impairs cellular life-expectancy and has been shown to have tissue and systemic repercussions including accelerated aging (Trifunovic and Larsson, 2008). In consensus, an accumulation of mitochondrial DNA (mtDNA) mutations, increased mitochondrial oxidative stress and a decrease in mitochondrial energy metabolism are all important contributors to aging (Lee and Wei, 2012). Mitochondria therefore play dominant roles in aging and marked effects of HAART upon mitochondria likely accelerate these effects. In this review we discuss how HAART is known to influence mtDNA integrity, alter mitochondrial morphology and function, induce oxidative stress, inflammation, and cell senescence, and how it is directly connected to aging symptoms and co-morbidities.

### DRUG INDUCED ACCUMULATION OF mtDNA DAMAGE

Because mitochondria contain their own DNA, mitochondrial genome integrity is essential for organelle function. The mtDNA encodes vital components of the mitochondrial respiratory chain and therefore damage to mtDNA is directly detrimental to energy metabolism and organelle fitness. Not surprisingly, cell senescence and aging are associated with an increase in the amount of damaged mtDNA. Additionally, accumulation of mutations in mtDNA is known to increase with age, and aberrant mtDNA replication contributes to premature-and-accelerated-aging phenotypes (Park and Larsson, 2011; Cline, 2012).

**FIGURE 2:**
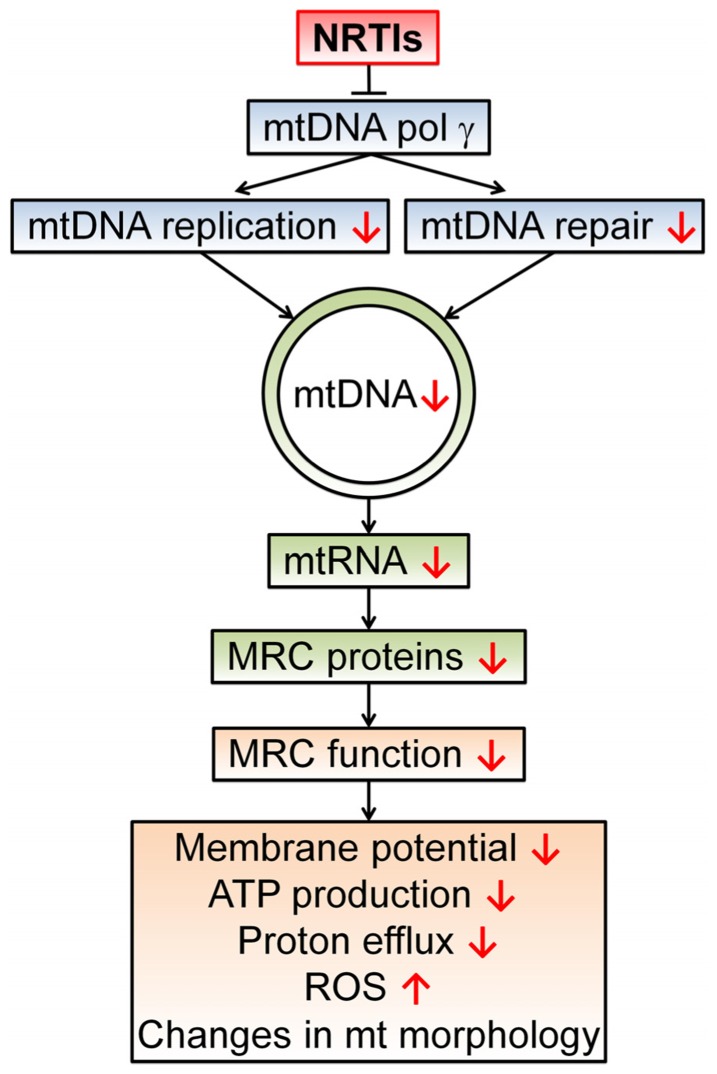
**The polymerase-γ theory**. NRTIs compete with endogenous nucleotides and nucleosides for transcriptase binding. Due to the surplus and high affinity of NRTIs for polymerase-γ, NRTIs are frequently incorporated into the new DNA strand which results in chain termination as they all lack the 3′-OH group necessary for phosphodiester bond formation in DNA strand elongation. This results in a reduced number of mtDNA molecules and possibly a reduction in mtDNA encoded proteins, essential components of the mitochondrial respiratory chain (MRC) complexes. In turn, this leads to disrupted electron transport through the MRC and a concomitant reduction in proton efflux, reducing the membrane potential and ATP production by the mitochondrion. This disturbed mitochondrial function can result in augmented ROS production and morphological changes. Disturbed mitochondrial function due to polymerase-γ inhibition has been proposed as a central mechanism for NRTI-induced adverse events (Lewis and Dalakas, 1995; Lewis et al., 2006).

DNA damage and unreliable replication can be induced by the backbone of antiretroviral therapy, namely NRTIs (Sundseth et al., 1996; Payne et al., 2011). NRTIs have been shown to inhibit the mitochondrial specific DNA polymerase-γ causing a decrease in mtDNA amount and quality. This discovery led to the theory of NRTI-induced toxicity commonly known as the “polymerase-γ theory” (**Figure 2**; Lewis and Dalakas, 1995). In short, a NRTI-induced decrease in mtDNA leads to malfunctioning of mitochondrial protein complexes and changes in respiration rate, decreased ATP production, a diminished mitochondrial membrane potential, and an escalation in ROS production (Lewis et al., 2006; Maagaard and Kvale, 2009). Besides direct inhibition of mtDNA replication, NRTIs also obstruct base excision repair and proofreading capabilities of polymerase-γ (Lim and Copeland, 2001; Lewis et al., 2003). Mice with impaired polymerase-γ proofreading ability show rapid accumulation of mtDNA mutations leading to disrupted mitochondrial function, a variety of aging phenotypes and early death (Trifunovic et al., 2004). Additionally, antiretroviral therapy likely hastens the expansion of pre-existing mutations in mtDNA as depleted mtDNA pools display accelerated digression from their original genetic content (Khrapko, 2011; Payne et al., 2011). The NRTI 3′-deoxy-3′-fluorothymidine (*alovudine* or FLT), known for its high toxicity, can cause DNA fragmentation and induce apoptosis (Sundseth et al., 1996). Interestingly, the NRTIs 3′-azido-3′-deoxythymidine (*zidovudine* or AZT) and 2′,3′-didehydro-2′,3′-deoxythymidine (*stavudine* or d4T) also disrupt telomerase maintenance and have telomere shortening effects, properties often related to cell senescence and aging (Strahl and Blackburn, 1994; Blasco, 2007).

During HAART it is very likely that NRTIs and PIs augment each other’s ability to steer the cell into premature senescence. This is especially the case when the “booster” PI *ritonavir* (RTV) is used in the HAART cocktail. RTV impedes the enzyme cytochrome P450-CYP3A4, which is responsible for the metabolism of xenobiotics, and therefore RTV induces an increase in intracellular drug concentrations in the patient (Zeldin and Petruschke, 2004). Interestingly, mtDNA damage has also been found to correlate with PI RTV use in human endothelial cell cultures in a dose-dependent manner (Zhong et al., 2002). Although mitochondria are the most important players in antiretroviral toxicities, outside these organelles PIs can cause accumulation of the farnesylated pro-senescence protein prelamin A. Prelamin A accumulation has been shown to cause genomic instability (Worman et al., 2009; Reddy and Comai, 2012). Furthermore, PI-induced prelamin A build-up is directly linked to increased oxidative stress and lipodystrophy-associated symptoms (Caron et al., 2007).

Mitochondrial DNA quantity and quality are important factors in mitochondrial functionality, and therefore cellular fitness, as mtDNA encode for vital components of the organelle’s respiratory chain complexes. Mitochondrial toxicity caused by NRTIs, however, does not necessarily follow the chronological steps of the polymerase-γ theory. Not every case of mtDNA depletion leads to changed expression levels or activity of mitochondrial respiratory chain proteins (Stankov et al., 2010). In addition, altered mitochondrial gene expression and impaired respiratory chain activity have been observed without mtDNA depletion (Mallon et al., 2005; Viengchareun et al., 2007). Expression profiles of mitochondrial mRNA possibly explain these occurrences as they have been shown to adjust, both in a peripheral blood mononuclear cell line and mice upon exposure to NRTIs. These adjustments likely reflect cellular adaptation to pressure on the mitochondrial transcriptional machinery (d’Amati and Lewis, 1994; Papp et al., 2008). In an elegant review, Apostolova et al. (2011a) show that mitochondrial toxicity of antiretroviral drugs goes beyond the polymerase-γ theory as disruption of many other mitochondrial mechanisms is also involved.

### OXIDATIVE STRESS

Reactive oxygen species, especially superoxide and hydrogen peroxide, are habitually produced in small quantities by mitochondria during oxidative phosphorylation. However, a decrease in, or malfunction of, mitochondrial proteins, due to diminished mtDNA for instance, can disrupt electron flow through the electron transport chain and cause increased ROS formation (Brand, 2010). Consequently, this increase in ROS can damage mitochondrial components, such as the electron transport complexes, and hence induce even more ROS production (Sastre et al., 2000). A fundamental feature of aging is a decline in mtDNA transcription and repair capacity which can lead to mitochondrial malfunction and set in motion a vicious cycle of enhanced ROS production (Desler et al., 2011). Interestingly, polymerase-γ is highly sensitive to oxidative damage and modification of its amino acid residues by oxidation brings about a decline in DNA-binding ability and polymerase activity (Graziewicz et al., 2002).

An increase in oxidative stress, observed as increased oxidant and reduced antioxidant levels in serum, has frequently been associated with HAART in patients (Mandas et al., 2009). Several studies conclude that symptoms of aging such as cardiovascular disease, lipodystrophy, and insulin resistance are all influenced by antiretrovirally induced ROS production (Day and Lewis, 2004; Caron-Debarle et al., 2010). A common side effect of AZT, namely cardiomyopathy, is likely caused by stimulation of ROS production in heart and endothelial mitochondria (Sutliff et al., 2002; Valenti et al., 2002). Prompt heart injury has even been ascribed largely to 2′,3′-dideoxycytidine (*zalcitabine* or ddC) induced ROS production, independent of mtDNA depletion or damage, a finding that emphasizes the impact of antiretroviral-induced ROS toxicity (Skuta et al., 1999). Increased oxidation of lipids, mtDNA and the major antioxidant glutathione (GSH), further relate AZT to skeletal muscle myopathy (de la Asunción et al., 1998). d4T is known to cause oxidative stress in human hepatoma cells and may underlie hepatic steatosis and lactic acidosis, which are often experienced by patients on HAART (Velsor et al., 2004). Thymidine analogs have additionally been shown to cause cell senescence through an increase in oxidative stress and induction of mitochondrial dysfunction in human fibroblast cell lines and in subcutaneous adipose tissue from HAART patients (Caron et al., 2008).

Protease inhibitors also have the potential to induce oxidative stress, although it is not always clear whether PI induced elevated ROS is produced at the mitochondrial level. The most clearly PI affected cell type is endothelial cells, although other cell types are also afflicted, and strong connections exist between drug toxicity and ROS production (Wang et al., 2007). RTV and *lopinavir* (LPV), two frequently prescribed PIs, can increase ROS production in human arterial endothelial cells (Lefèvre et al., 2010) and are known to induce ROS through a perturbed mitochondrial function in cardiomyocytes (Deng et al., 2010). *Indinavir* (IDV) and *nelfinavir* (NFV) have been shown to elicit ROS production in skin fibroblast cultures *in vitro* and in patients’ adipose tissue *in vivo* (Viengchareun et al., 2007). IDV and NFV have furthermore been shown to cause ROS production in human aortic endothelium and are thus involved in recruitment of mononuclear cells and exacerbation of inflammation, prerequisites for vascular complications (Mondal et al., 2004). Additionally, treatment with IDV or NFV was shown to cause increased mitochondrial ROS production and premature senescence in skin fibroblasts (Caron et al., 2007), and an IDV and AZT combination induces ROS mediated apoptosis in human brain microvascular endothelial cells (Manda et al., 2011). Short-term treatment of NFV increases ROS generation and diminishes levels of GSH and the detoxification enzyme superoxide dismutase in a pancreatic insulinoma cell line (Chandra et al., 2009). Moreover, NFV has been linked to adipocyte insulin resistance through oxidative stress induced apoptosis and necrosis (Vincent et al., 2004; Ben-Romano et al., 2006), which is noteworthy as the anti-apoptotic properties of PIs in a low-dose have been documented (Badley, 2005). *Saquinavir* (SQV) however, was shown to cause apoptosis in human umbilical vein endothelial cells via higher levels of ROS production (Baliga et al., 2004). SQV, IDV, NFV, and RTV also elevate ROS in cerebral endothelial cells and interfere with proper blood brain barrier maintenance. Therefore, these PIs conceivably play a significant role in antiretroviral-induced neurological symptoms and could also increase viral entry into the central nervous system (Grigorian et al., 2008). Collectively, these results indicate that oxidative stress is a powerful driving force behind antiretroviral-induced toxicity and has important roles in premature-and-accelerated-aging symptoms (Blas-Garcia et al., 2011).

### ALTERED MITOCHONDRIAL MORPHOLOGY AND FUNCTION

Mitochondria are no longer considered as static spherical bodies, but highly dynamic organelles that readily fuse, divide, propagate, and diminish according to cellular requirements. Mitochondrial morphology plays an essential role in mtDNA rescue, protein quality control, and cell survival (Bess et al., 2012; Shutt and McBride, 2012). Certain distinct morphological changes in mitochondrial structure and organization are therefore considered indicators of aging in worms, mice, and humans (Jendrach et al., 2005; Yasuda et al., 2006). Specifically, mitochondria of aged individuals are often swollen and their structures contain less villous cristae, while the mitochondrial network is frequently disrupted (Sastre et al., 2000). Mitochondrial function, especially respiration and ATP production, has been demonstrated to decline with age and even be an important mediator of senescence (Desler et al., 2012). Energy deficiency can cause a broad range of metabolic and degenerative diseases including aging (Wallace et al., 2010). Mitochondrial processes for example play important roles in adipocyte differentiation and function, which in turn influence a wide array of homeostatic processes including insulin sensitivity and lipid accumulation (Caron-Debarle et al., 2010). Changes in mitochondrial structure and function are known to occur in age-associated disorders such as Parkinson’s disease, sarcopenia and metabolic diseases, including heart-disease and diabetes mellitus (Desler et al., 2012; Galloway and Yoon, 2012).

Not surprisingly then, antiretroviral drugs are found to alter mitochondrial morphology and function, although specific mechanisms and the chronology of these events remain to be fully unraveled. Electron microscopy of AZT-treated striated skeletal muscle from rats, and AZT-, ddC-, and 2′,3′-dideoxyinosine (*didanosine *or ddI)-treated human hepatocytes show widespread mitochondrial swelling with poorly organized cristae (Lewis et al., 1992; Pan-Zhou et al., 2000). Muscle biopsies from AZT-treated patients give similar results with striking variations in mitochondrial size, shape, and network organization (Pezeshkpour et al., 1991). AZT and d4T induce a rapid increase in mitochondrial proliferation in human fibroblasts (Caron et al., 2008), and their combination with or without IDV increase mitochondrial mass in both white and brown murine adipocytes (Viengchareun et al., 2007). Individual exposure of HeLa cells to NFV, RTV, and SQV caused fragmentation of the mitochondrial network and decreased mitochondrial number and volume (Roumier et al., 2006).

Mitochondrial fusion, fission, and autophagy have important roles in mitochondrial maintenance, specifically in protection against persistent mtDNA damage (Chen et al., 2010; Bess et al., 2012). Therefore, altered mitochondrial morphology might be considered a compensatory mechanism to help preserve mitochondrial functions. Increased proliferation for example, may be an attempt of mitochondria to recover mtDNA and increase functional capacity under pressure (Lee and Wei, 2005). However, evidence exists that the newly formed mitochondria could be non-functional (Caron et al., 2008). Mitochondrial autophagy on the other hand, has been interpreted as a protective mechanism against NNRTI *efavirenz*-induced respiratory chain malfunction (Apostolova et al., 2011b).

Murine adipocytes exposed to AZT, d4T, and/or IDV displayed impaired mitochondrial function as measured by lower respiration rate and decreased ATP production (Jiang et al., 2007; Viengchareun et al., 2007). AZT is also known to competitively inhibit the ADP/ATP antiporter in rat heart mitochondria and thus could contribute to the ATP deficiency syndrome witnessed in patients (Valenti et al., 2000). Cells with diminished oxidative phosphorylation shift to glycolysis for their energy demands which results in accumulation of lactate and, if left untreated, can cause lactic acidosis. AZT-, d4T-, or ddC-treated human hepatoma cells show increased lactate concentrations and, in some cases, decreased activity of mitochondrial respiratory chain complexes (Velsor et al., 2004). An analysis of mitochondrial genes in adipose tissue and monocytes from HIV-negative subjects receiving dual NRTI therapy revealed a significant decrease in mitochondrial respiratory chain component expression (Mallon et al., 2005). AZT and IDV have additionally been found to suppress membrane potential and cause apoptosis in blood–brain barrier endothelial cells (Manda et al., 2011). Moreover, PI-induced mitochondrial effects are typically related to an altered membrane potential (Apostolova et al., 2011a). A randomized, double-blind, placebo-controlled study found that short-term AZT exposure reduced mitochondrial function and insulin sensitivity in non-infected participants (Fleischman et al., 2007). Additionally, a randomized clinical trial in non-symptomatic antiretroviral-naïve patients showed that long-term exposure to PIs or NNRTIs is associated with disrupted glucose transport as well as disrupted lipid metabolism with increased insulin resistance (Shlay et al., 2007). In conclusion, antiretroviral therapy has frequently been implicated in metabolic diseases as a result of mitochondrial dysfunction (Caron-Debarle et al., 2010) and mitochondrial impairment is found in the absence of HIV infection.

## IS HAART INVOLVED IN IMMUNOSENESCENCE?

With the success of HAART in viral suppression the question arises whether HIV-1 is the sole plausible cause for immunosenescence in HIV-treated patients. HAART, which is taken daily for lifelong periods, is probably also responsible for immune system malfunction. Besides that various antivirals have been shown to induce inflammatory signals (Mondal et al., 2004; Lagathu et al., 2007; Lefèvre et al., 2010), senescent cells have also been shown to change their phenotype, secreting proinflammatory cytokines and contributing to systemic low-grade inflammation (Freund et al., 2010). The systemic exposure and relatively high concentration of antiretrovirals undoubtedly affects all cell types, immune system cells included. The direct relationship between antiretroviral drugs and inflammation needs to be addressed further.

Hematopoietic progenitor and lymphoblastoid cell toxicity of NRTIs may explain immune cell depletion independent of inflammation (Faraj et al., 1994; Sundseth et al., 1996; Sharma, 2010). Moreover, a decline in mitochondrial genetic integrity in hematopoietic progenitor cells could also explain continued immune dysfunction upon cessation of therapy. mtDNA levels do recover in patients after discontinuation of HAART (Côté et al., 2002), but due to generation of somatic mutations by antiretrovirals and ROS, it is likely that replenished mtDNA harbors mutations predisposing the recuperating mitochondria to continued dysfunction.

## A SUITABLE MODEL SYSTEM TO STUDY PREMATURE AND ACCELERATED AGING

Many questions remain unanswered in the antiretroviral drug field. Most HIV-1-infected individuals use a numerous combination of antiretroviral drugs from two or more different classes, making singular drug impacts difficult to assess. Furthermore, patient populations are diverse and administered drug cocktails as well as research methods are often dissimilar (Fisher and Cooper, 2012). There are multiple *in vivo *and *in vitro *model systems in use to study drug toxicity, however complex systems are time consuming and expensive and they do not permit straightforward analysis. Undeniably, the lack of a good model system has hampered consistent and coherent research into specific effects of antiretroviral therapy.

The nematode *Caenorhabditis elegans* has proven itself to be one of the most versatile model organisms for the elucidation of molecular pathways implicated in many human diseases, including those of mitochondria and aging (Culetto and Sattelle, 2000; Markaki and Tavernarakis, 2010). Aging in *C. elegans* is entirely post-mitotic, reflecting the gradual loss of function in somatic cells as they grow old. Although limited, this model system can also help researchers dissect tissue- and compartment-specific effects. *C. elegans* normally has a relatively short lifespan of two weeks, enabling researchers to rapidly assess the effects of different mutations or treatments on lifespan. Mitochondrial research in *C. elegans* has given us many insights into the genetic regulation of aging and mitochondrial function, and it has provided us with a vast array of mutants to study these effects (Tsang and Lemire, 2003; Addo et al., 2010; Bratic et al., 2010). Not only is *C. elegans *a very practical system, this nematode has also been used to study drug-specific impact on mitochondria (Zubovych et al., 2010). With this knowledge we can use *C. elegans *to quickly evaluate the effects of individual antiretroviral drugs, not only on mitochondrial function directly, but in relation to organism genetics, physiology, and longevity.

*Caenorhabditis elegans *has successfully been used to elucidate specific effects of NRTIs on physiology and longevity (R. de Boer and H. van der Spek, personal communication). *C. elegans *demonstrated that NRTIs decrease mtDNA copy number, disrupt both structure and function of mitochondria and shorten the average lifespan. Using oxygen consumption as a measure of mitochondrial function, NRTIs were shown to induce a rapid decrease in mitochondrial fitness. These findings compare well to earlier studies wherein abnormal mitochondrial respiratory activity was correlated with altered expression or deficiency in various respiratory chain complexes (Pan-Zhou et al., 2000; Caron et al., 2008). Additionally, *C. elegans* mitochondria showed signs of increased mass, fragmentation, and disrupted organization, as is typically found in HAART treated patients (R. de Boer and H. van der Spek, personal communication).

## CONCLUSION

**FIGURE 3:**
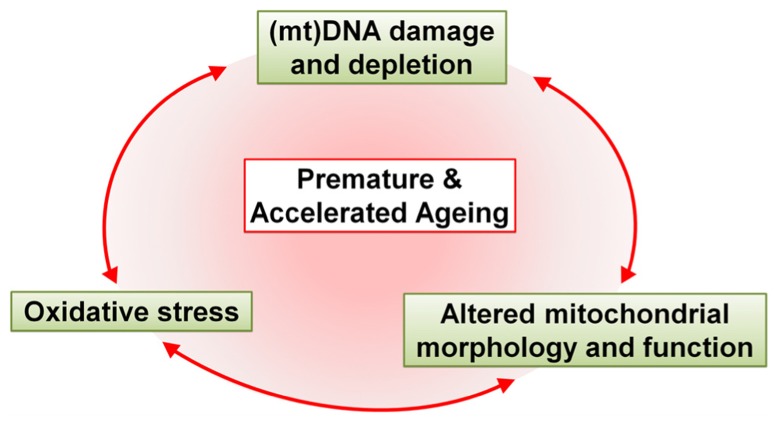
**Schematic representation of the major effects of antiretroviral drugs that drive premature and accelerated aging**. Antiretroviral drugs cause mtDNA damage and depletion, oxidative stress and altered mitochondrial morphology and function. These alterations in the mitochondria contribute, either alone or in unison, to premature and accelerated aging in HAART-treated patients.

With the increase in life expectancy it has only recently become clear that HIV-1 patients are suffering from symptoms of aging ahead of time. In this review we postulate that strong correlations exist between antiretroviral drug-induced mitochondrial toxicity and premature and accelerated aging (**Figure 3**). However, there are a number of questions that remain unanswered, simply because we do not fully understand the effects of antiretroviral drugs individually, let alone in combination.

For example, questions remain about mitochondrial toxicity of NRTIs beyond the polymerase-γ theory. Various cellular transport systems interact with NRTIs and once inside the cell NRTIs are actively phosphorylated from their pro-drug form (Lewis et al., 2003). Changes in cellular thymidine kinase kinetics by interaction with NRTI thymidine analogs have been linked to cardiomyopathy and lipodystrophy (Apostolova et al., 2011a). Additionally, the occasionally divergent relationship between mtDNA copy number and respiratory chain protein levels needs to be explained. The influence NRTIs have on gene expression could give us insight into cellular adaptation to antiretroviral drugs. Alleviation of oxidative stress could prove an easy way to improve the well-being of patients and delay the detrimental effects of antiretroviral drugs. Interestingly, most of the above mentioned ROS complications have experimentally been found to lessen upon co-administration of antioxidant compounds. Antioxidant- or mitochondria-directed supplementation may therefore benefit HAART patients, although thorough research remains to be done before any definitive advice can be given to patients (Neustadt and Pieczenik, 2008).

We propose the use of *C. elegans* as a model system to study the effects of antiretroviral therapy on premature and accelerated aging. Using *C. elegans *we can begin to study the effects of specific genetic backgrounds on HAART toxicities. Effects of HAART seen in the nematode can direct more specific research into human conditions. In addition, *C. elegans* could provide an easy platform, not just for toxicity studies of various antiretroviral drugs, but also to screen for suitable compounds that neutralize toxic effects of HAART, which remains crucial as long as total HIV eradication is not possible. Combining these genetic and toxicology approaches we can initiate research leading to efficient, personalized, anti-HIV treatment in humans.

## Conflict of Interest Statement

The authors declare that the research was conducted in the absence of any commercial or financial relationships that could be construed as a potential conflict of interest.
